# Particulate vs monolithic aerogel linings in capillaries for Raman signal gain of aqueous solutions

**DOI:** 10.1007/s00216-025-06008-6

**Published:** 2025-07-18

**Authors:** Felix Spiske, Andreas Siegfried Braeuer

**Affiliations:** Institute of Thermal, Environmental and Resources’ Process Engineering (ITUN), Technische Universität Bergakademie Freiberg (TUBAF), 09599 Freiberg, Germany

**Keywords:** Aerogel-lined capillary, Liquid core waveguide, Monolithic aerogel-lined capillary, Raman spectroscopy, Particulate aerogel-lined capillary, Liquid core waveguide capillary cell

## Abstract

We compare particulate versus monolithic silica aerogel-lined capillaries as liquid core waveguides for Raman signal gain of liquid aqueous samples. The manufacturing process of monolithic linings, which aims to address the limited robustness of particulate aerogel linings, is discussed in detail and compared with that of particulate linings. The linings of both capillary types are characterized using scanning electron microscopy. Raman signal gain of pure water and aqueous solutions is evaluated for each type. The aqueous solutions are samples drawn at different times from the oxidative conversion of glycerol to formic acid (OxFA process). It is analyzed whether the Raman signal gain provided by aerogel-lined capillaries is affected by the property changes in this exemplary industrial process caused by the ongoing chemical reaction. Finally, the mechanical robustness of the linings and the stability of the Raman signal gain are tested under rapid and repeated capillary purge in both types.

**Graphical Abstract:**

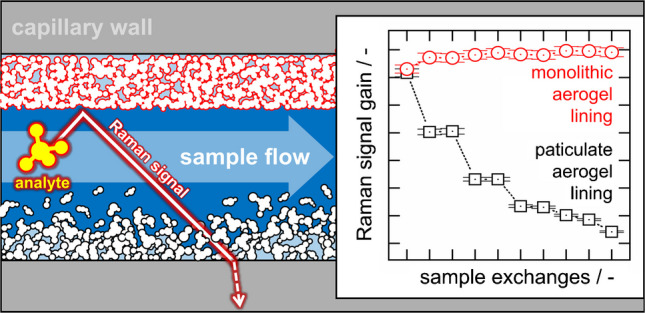

## Introduction

Real-time monitoring of chemical processes in aqueous solutions is increasingly carried out using Raman spectroscopy (RS). Unfortunately, the small Raman scattering cross-section requires high excitation laser power in order to lift the Raman signal above the noise. However, in ex-zones of chemical plants, the laser power is strictly limited, creating a strong need for technologies to enable a Raman signal gain without increasing the laser power [1, 2]. Next to multi-pass Raman cells [3–5] liquid core waveguides (LCWs) are an efficient solution for Raman signal gain [6–8]. The Raman signal gain of LCWs is simply based on the enhancement of the probed volume. As shown in Fig. 1 in [9], a LCW is a capillary which guides light—in our case, the excitation laser as well as the Raman signal—via total internal reflection, because the capillary is lined with a material featuring a lower refractive index (RI) than the liquid sample in the capillary core. As the critical angle of total internal reflection according to Snell’s law is a function of the difference between the refractive indices of the liquid sample and the lining, silica aerogels with air-like refractive indices of 1.01–1.2 are highly qualified to be used as lining materials in LCWs [10–12].

In previous publications [9, 13], we demonstrated that capillaries lined with hydrophobic aerogel particles (**P**articulate **A**erogel-**L**ined **C**apillaries, PALCs) gain Raman signals of aqueous solutions by more than one order of magnitude, with no background signals from the lining itself. Due to the hydrophobicity of the lining, the physical contact between the lining and the aqueous sample in the core is quasi-avoided. Therefore, the silica aerogel lining promises high resistance against fouling, scaling, and corrosion. A key advantage of the PALC manufacturing is its efficiency and reproducibility. Unfortunately, the particles in a PALC aerogel lining only adhere to each other through physical interactions. As a result, the brittle lining tends to crack at thicknesses above around 1000 µm, and aerogel particle agglomerates can detach from the lining at high shear stress when, for example, samples are pumped rapidly through the PALC’s hollow/liquid core. This causes irreversible damage to the lining of the PALC, clogging of PALCs by the detached agglomerate flakes, contamination of the process with silica particles, and ultimately costly production downtime. In order to address this challenge, we here present a method for producing capillaries lined not with a layer of agglomerated aerogel particles but with a layer that consists of a single aerogel monolith (**M**onolithic **A**erogel-**L**ined **C**apillaries, or MALCs). We characterize the monolithic linings and compare their properties and manufacturing process to those of PALCs. Additionally, we analyze whether the Raman signal gain provided by aerogel-lined capillaries is affected by the property changes in a sampled industrial process medium caused by the ongoing chemical reaction. Finally, we assess the robustness of both MALCs and PALCs during frequent and rapid purging with samples.

## Materials and methods

### Materials

The aqueous solution of sodium silicate (CAS-No. 1344–09-8), as well as cyclohexane (CAS No. 110–82-7), ethanol (CAS-No. 64–17-5) and the aqueous ammonia solution (6N) (CAS No. 1336–21-6) were purchased from VWR chemicals (Darmstadt, Germany). The Amberlite® IR-120 (H) ion exchange resin (CAS-No. 78922–04-0) and tetraethyl orthosilicate (TEOS) (CAS-No. 78–10-4) were purchased from MERCK (Darmstadt, Germany). Trimethyl chlorosilane (TMCS) was purchased from Thermo Scientific (Waltham, MA, USA). The fused silica capillaries with inner diameters of 700 µm were purchased from MOLEX (Lisle, IL, USA).

### Preparation and characterization of the aqueous samples

In order to analyze how the Raman signal gain provided by MALCs and PALCs is affected by chemical and physical property changes in a sampled process medium, four samples were taken from the exemplary OxFA process [14], from start to endpoint, thus reflecting the evolution of the properties of the process medium throughout the full process. The oxidative conversion of aqueous glycerol to formic acid (OxFA process) was carried out batchwise at OxFA (Scheßlitz, Germany). At $t=0, 7, 20, \text{and}\; 60\; \text{h}$ after process start samples were taken from the reactor and are in the following and in Table 1 referred to as samples #1, #2, #3, #4. Since the compositions of the samples must not be disclosed, we here report the progress variable $C$ calculated as the ratio of the amount of substance of formic acid ${n}_{FA}(t)$ in the particular sample and the amount of glycerol presented in the reactor at the beginning of the process ($t=0$ h). Dilutions of the samples #1–4 were prepared by filling up 65.0 mL and 12.5 mL of the samples to 100 mL. The respective diluted samples are referred to as 65% vol or 12.5% vol.

**Table 1:** Specification of the samples taken from the OxFA process

Sample	Sampling time/h	C/-
#1	0	0
#2	7	0.25
#3	20	0.40
#4	60	0.52

### Determination of the Raman signal gain achieved by an aerogel-lined capillary

Raman spectra of liquid samples filled in a cuvette or in the hollow core of a PALC or MALC were measured using the setup depicted in Fig. 5 in [9], in which the laser source was a Cobolt Samba 1000 (HÜBNER Photonics GmbH, Kassel, Germany) and the spectrometer a QEPro (Ocean Optics, Ostfildern, Germany). The Raman signal gain

1 $$G=\frac{{A}_{ALC}}{{A}_{CUV}}$$

is the ratio of Raman signal intensities detected from the sample inside the MALC or PALC (${A}_{ALC}$) and from the sample outside the MALC or PALC but inside the cuvette (${A}_{CUV}$). Only for the measurement inside the PALC or MALC (${A}_{ALC}$) the probed volume is increased due to total internal reflection, while the measurement outside the PALC or MALC but inside the cuvette (${A}_{CUV}$) serves as reference. The intensities ${A}_{ALC}$ and ${A}_{CUV}$ are determined from the recorded Raman spectra as the integrals of the Raman signal intensity between 2900 and 3800 cm^−1^.

### Scanning electron microscopic characterization of aerogel linings

The measurement of the thickness of the aerogel linings and the characterization of their topography was performed as described in [9] using a XL30 ESEM scanning electron microscope (PHILIPS, 22335 Hamburg, Germany).

### Estimation of the density and the refractive indices of monolithic and particulate silica aerogels

**Fig. 1:**
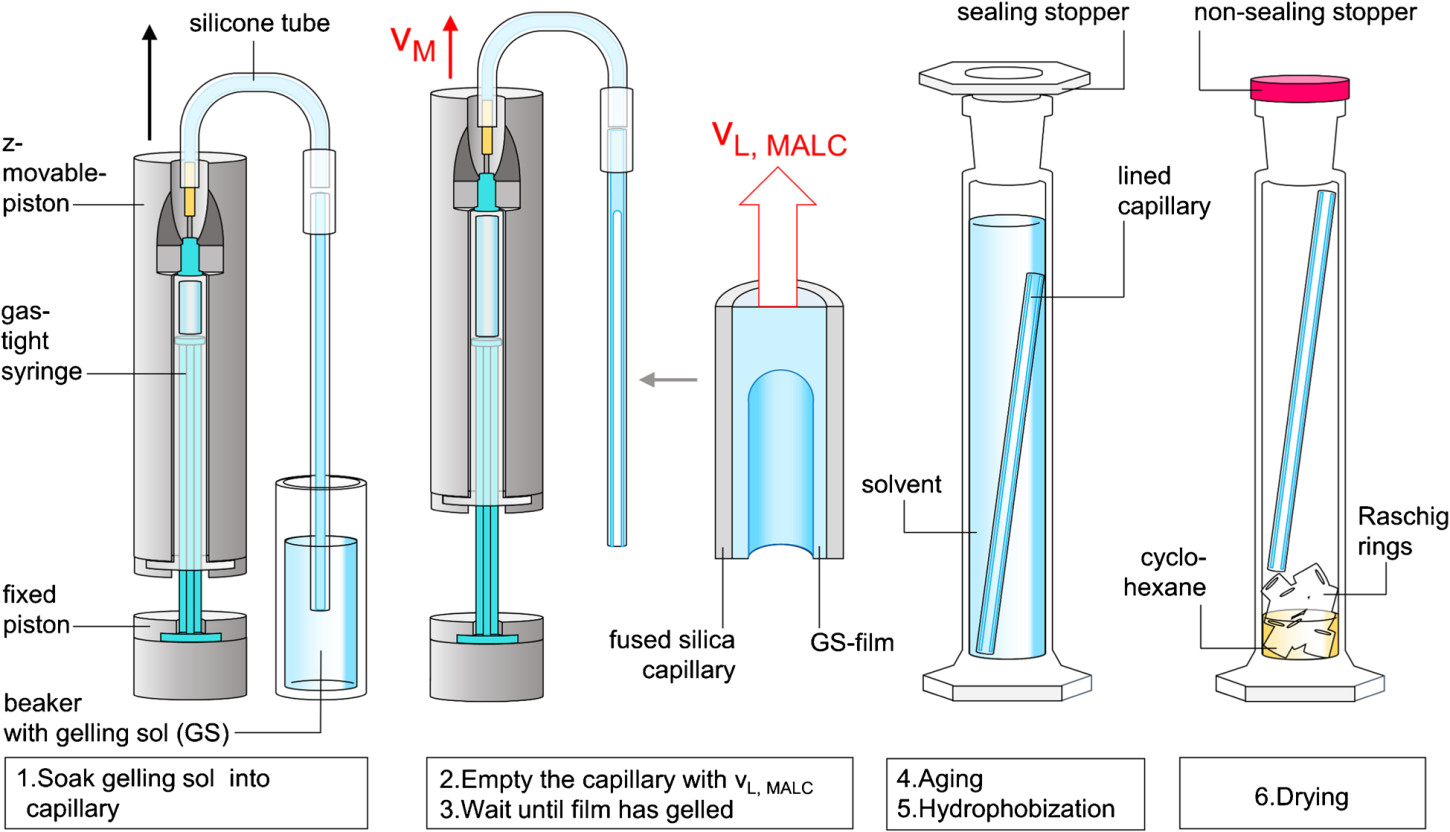
The manufacturing of a MALC involves lining a capillary with a gelling sol (GS), followed by aging, hydrophobization, and drying of the monolithic lining

In order to estimate the density of a PALC aerogel lining, the coating dispersion (produced according to [9]) was stored for several days at 50 °C until the ethanol was evaporated completely, leaving only the hydrophobized silica aerogel particles, of which the raw density ${\rho }_{PALC}$ was then measured using Hg intrusion (Poremaster from 3P INSTRUMENTS). For the estimation of a MALC lining density, the gelled gelling solution from the beaker (shown in Step 1 in Fig. 1) was crushed with a spatula, then aged, hydrophobized and dried. The raw density of the resulting hydrophobic aerogel flakes ${\rho }_{MALC}$ was also measured using Hg intrusion. Finally, the refractive indices

2 $${n}_{MALC\; or\; PALC}=1+\frac{{n}_{{SiO}_{2}}-1}{{\rho }_{{SiO}_{2}}}\bullet {\rho }_{MALC\; or\; PALC}$$

of the silica aerogel linings of the MALCs or PALCs were calculated with the density of amorphous silicium dioxide ${\rho }_{{SiO}_{2}}=2.19\; g\bullet {cm}^{-3}$ and the refractive index of silicium dioxide ${n}_{{SiO}_{2}}=1.45$, according to [15], where the derivation of Eq. 2 is also shown.

### Measuring the contact angle of liquid samples on a silica aerogel surface

The measurement of the contact angle (CA) between a hydrophobic aerogel surface and a 2.0 µL droplet of a liquid sample was conducted using a KRÜSS DSA25S drop shape analyzer. The hydrophobic silica aerogel surface was produced by dip-coating a glass microscope slide with the PALC-coating dispersion, produced according to the recipe described in [9] and summarized in Table 2. During dip-coating, the slides were pulled from the coating dispersion with a velocity of 150 mm∙min^−1^.

### Measuring the interfacial tension between the liquid samples and air

The interfacial tension between the liquid samples and air was measured using the pendant drop method at a KRÜSS DSA25S drop shape analyzer at $T=20^\circ C$. The density of a liquid sample, which is required for the derivation of the interfacial tension, was the mean value of five individual measurements. Each individual measurement was made by taking 5000 µL from water or the OxFA sample, weighing it and dividing mass by volume. We took $0.00129\; g\bullet {cm}^{-3}$ as the density of the ambient air.

## Results and discussion

### Manufacturing and characterization of particulate and monolithic silica aerogel linings

The production of a monolithic silica aerogel-lined capillary (MALC) requires the silica monomers to gel in a liquid film on the inner surface of the wall of a fused silica capillary. On the contrary, during the production of a particulate aerogel-lined capillary (PALC), the silica monomers gel in liquid methanol at 50 °C in a closed beaker over 48 h (see step 1 in Table 2), before the monolithic product is then processed into an ethanolic coating dispersion (CD) of hydrophobic silica particles (steps 2–4 in Table 2).

**Table 2:** The four basic steps for manufacturing a particulate aerogel–lined capillary (PALC) [9]. (CD: coating dispersion)

Step	Procedure	Product	Pore fluid
1	Gel tetramethyl orthosilicate (TMOS) in methanol at 50 °C in a closed beaker over a period of 48 h	Hydrophilic alcogel monolith	Methanol, NH_3_ (aq.)
2	Crush and hydrophobize monolith and exchange pore fluid with ethanol	Crushed hydrophobic alcogel	Ethanol
3	Disperse crushed hydrophobic monolith in a defined amount of ethanol using ultrasonic dispergator	PALC-coating dispersion (CD)	Ethanol
4	Line a fused silica capillary with the CD and dry the lining by conveying air though the CD-lined capillary	PALC	Air

The high volatility of methanol makes it challenging to manufacture the MALCs with the same chemistry we used for the manufacturing of PALCs. The TMOS-based sol–gel synthesis relies on the presence of the highly volatile methanol. If the TMOS-based solution was coated as a thin film on the inner surface of a capillary, significant amounts of methanol will have evaporated before gelation has even begun. Therefore, in MALC production, we perform the sol–gel synthesis in a low volatile aqueous solution of free silicic acid deposited as gelling film on the inner surface of a capillary wall. Using water as solvent, there is quasi no evaporation of the solvent during gelation which advantageously is completed in just a few minutes at pH = 5–6 [16, 17] and room temperature. However, aqueous silica solutions cannot be purchased as they intrinsically gel over time and therefore must be freshly synthesized from sodium silicate via ion exchange. Thus, fabrication of a MALC starts with mixing 80 mL deionized water with 20 mL aqueous solution of sodium silicate, followed by adding acid ion exchange resin until pH = 2–3. The resin is then separated by filtration, and the filtrate is divided into as many small beakers as MALCs are to be produced. At pH = 2–3, the gelation of the silicic acid monomers occurs slowly, but can be accelerated by raising the pH to 5–6. Therefore, in the next step a 1 M ammonia solution is added dropwise into a stirred beaker of 5 mL aqueous solution of silicic acid at pH = 2–3 until pH = 5–6 creating a gelling sol (GS). Then, the bottom of a fused silica capillary is immediately immersed in the gelling sol contained inside the beaker (Step 1 in Fig. 1), with the top connected to a gas-tight syringe (1.0 mL volume, 4.38 mm inner diameter) clamped in a self-engineered syringe pump (based on a modified tensile testing machine INSPEKT mini, Hegewald&Peschke Meß- und Prüftechnik GmbH, Nossen, Germany). In order to increase the reproducibility of the MALC manufacturing process, we always waited exactly 1 min after step 1 before carrying out step 2. This break is defined as “holding time”. Then, the syringe is pulled apart at ${v}_{M}$=100 mm∙min^−1^ filling the capillary with the gelling sol. Once filled, the bottom of the capillary is removed out of the gelling sol pool inside the beaker, and the gelling sol is conveyed completely out of the capillary into the syringe (Step 2 in Fig. 1) at a mean flow velocity, called the lining velocity ${v}_{L,\; MALC}$ (specified in mm∙s^−1^) which is calculated based on the inner diameters of capillary and syringe and the velocity ${v}_{M}$, at which the barrel and the plunger of the syringe were pulled apart. However, when conveying the gelling sol out of the capillary, a film of the gelling sol remains on the inner surface of the capillary. The gelling sol wets the inner surface of the capillary.

The gelation of the micro-thin transparent gelling sol-film in the capillary is not visible. But the gelation of the bulk gelling sol that remained in the beaker is visible. Assuming the gelation rates in the gelling sol-film and beaker are the same, the gelling sol-lined capillary is kept hanging vertically at the silicone tube until gelation in the beaker is finished (step 3 in Fig. 1). The gelation took about 8 min after the adjustment of the pH to 5–6. Once the gelling sol-film has gelled, the capillary (now having a lining of hydrophilic monolithic silica hydrogel) is immersed in an aging solution (= TEOS and ethanol, 7:3 by volume) at 70 °C for 8 h, allowing TEOS to condense with the silica network and strengthen it (step 4 in Fig. 1). Next, the capillaries are stored at 65 °C for 6 h in ethanol. The ethanol is refreshed after 2 and 4 h. This process of storing and refreshing is then repeated with cyclohexane instead of ethanol, in exactly the same manner. The cyclohexane is replaced with a hydrophobization solution (= TMCS and cyclohexane, 6:94 by volume), in which the capillary is stored at 65 °C for 3 h. Afterwards, the capillary and cylinder are emptied, cyclohexane and glass packing (e.g., Raschig rings) are added, and the capillary is placed on the dry glass packing, but not in the liquid, as shown in step 6 in Fig. 1. The cylinder is stored at room temperature for 24 h with the stopper half closed. In order to dry any remaining liquid, the capillary is then removed and heated at 220 °C for 1 h, resulting in the desired monolithic aerogel-lined capillary (MALC).

**Fig. 2:**
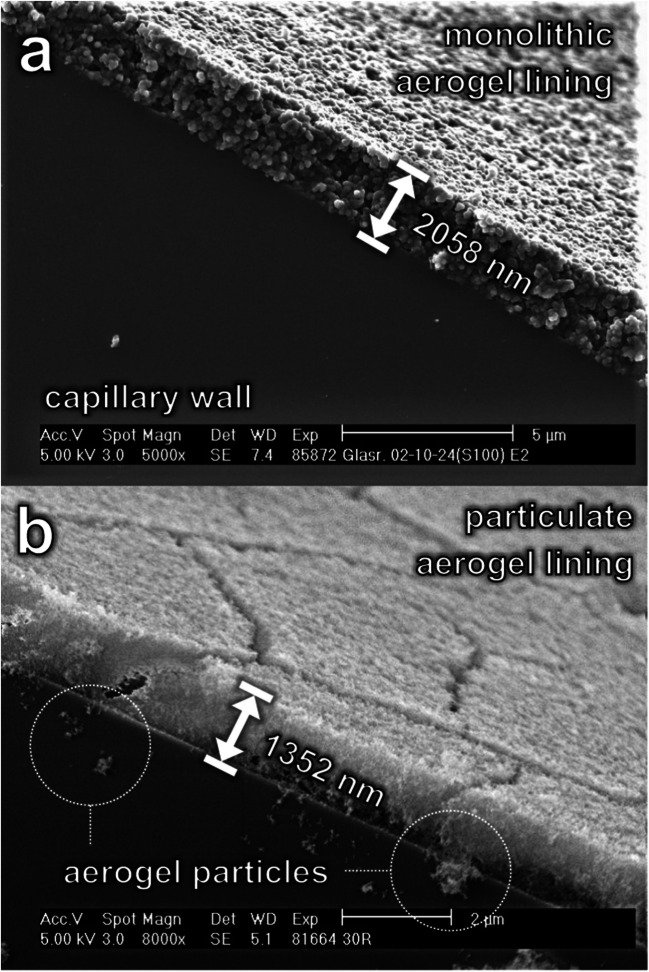
Scanning electron microscope images of **a** the monolithic aerogel lining of a MALC and **b** the cracked particulate aerogel lining of a PALC with loose aerogel particles detached from the lining

In comparison, the key difference between PALCs’ and MALCs’ manufacturing is that for PALCs, an ethanolic dispersion of hydrophobic silica particles (the coating dispersion “CD”) is used for lining the capillary (see step 4 in Table 2), while for MALCs a gelling sol is used (see step 1 in Fig. 1). The lining velocities applied for lining a capillary are designated as ${v}_{L,\; MALC}$ and ${v}_{L,\; PALC}$, respectively. However, in PALC production, hydrophobization occurs before lining the capillary (see step 3 in Table 2), while in MALC production, it happens after the lining process (see step 5 in Fig. 1). However, it should be mentioned that the manufacturing of MALCs is more complex than that of PALCs. This means that in the case of PALC manufacturing, a large batch of crushed hydrophobic monolithic alcogel (the product of Step 2 in Table 2) can be produced once, stored indefinitely long, and when needed, a desired amount can be taken, easily processed into a PALC-coating dispersion (using an ultrasonic dispergator), and used for the quick lining of a capillary. On the contrary, producing a single batch of MALCs requires the fresh preparation of a silicic acid solution via acidic sodium ion exchange of an aqueous solution of sodium silicate, as the silicic acid solution cannot be stored without gelling. After film coating, aging, hydrophobization and drying follows for every single batch. However, electron microscopic images of MALC and PALC aerogel linings (see Fig. 2) highlight the benefit of the higher manufacturing effort of MALCs. While the monolithic aerogel lining of the MALCs (see Fig. 2a) shows no tendency to crack at a thickness of around 2.1 µm, the lining of the PALC, which is only 1.3 µm thick, is riddled with cracks. Figure 2b also shows that loose aerogel particle agglomerates can detach from the particulate lining. It is also noticeable that the particulate lining is made of particles, which are significantly smaller than the beads the monolithic lining is made of.

**Fig. 3:**
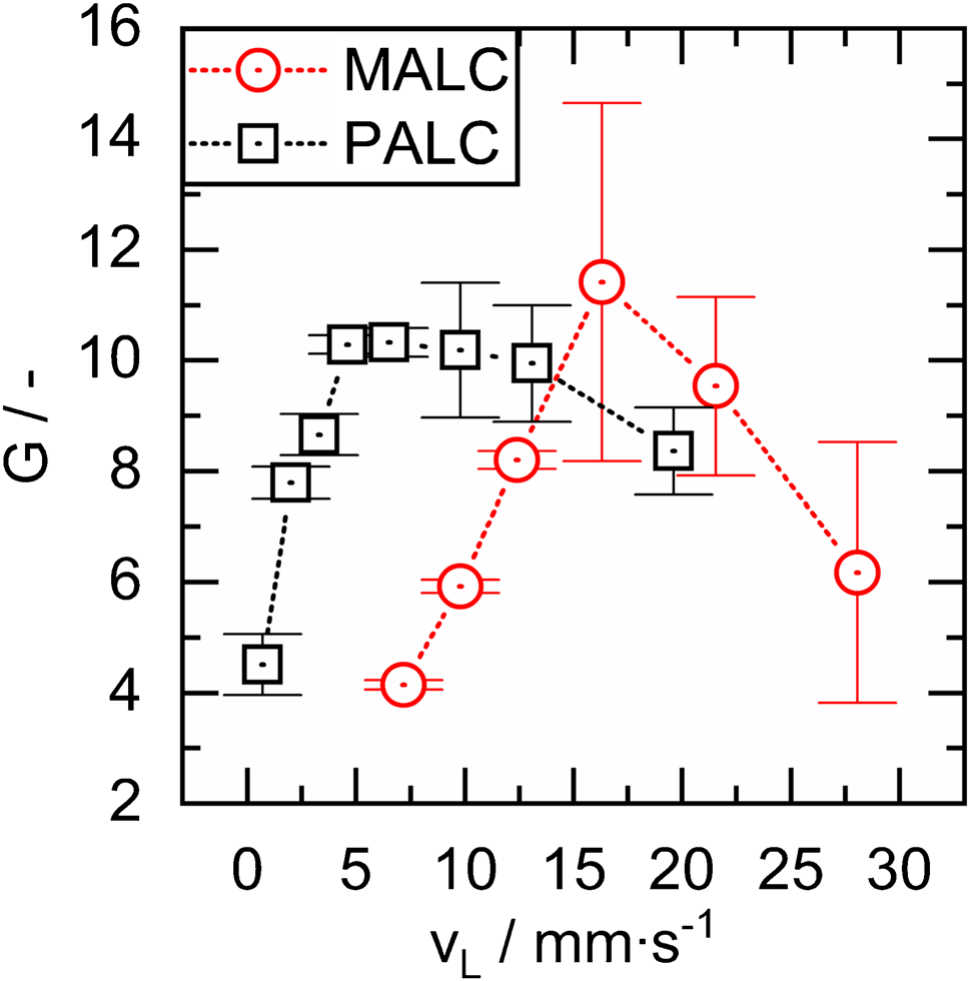
Raman signal gain G of pure liquid water obtained from PALCs and MALCs fabricated at lining velocities in the range from 2.0 to 28.1 mm∙s^−1^ (the data for the PALCs are taken from [9])

In [9], we demonstrated that the Raman signal gain (of pure liquid water) achieved by a PALC (20 cm length, 700 µm inner diameter) can be controlled by adjusting the lining velocity ${v}_{L,PALC}$, and now we show in Fig. 3 that this also applies to the manufacturing of MALCs of equal dimensions and geometry (the values $G$ are the mean values from three individual measurements and the error bar shows the single standard deviation).

It is also noticeable that higher lining velocities in MALCs are needed than in PALCs in order to achieve the same $G$ (note the rightward shift of the red curve). This is likely due to the lower viscosity of the MALC-gelling solution, resulting in a lower thickness (and hence a lower $G$) of the MALC lining, if ${v}_{L,MALC}={v}_{L,PALC}={v}_{L}$. Moreover both, PALCs and MALCs, exhibit $G({v}_{L})$-curves with optima at ${v}_{L,PALC}\approx 10\; mm\,{\bullet\; s}^{-1}$ and ${v}_{L,MALC}\approx 16\; mm\,{\bullet\; s}^{-1}$, respectively. The fact that both optima are $G\approx 11$ corresponds well with the derived similar refractive indices of the aerogel linings ${n}_{PALC}\approx 1.05$ and ${n}_{MALC}\approx 1.04$. For PALCs the occurrence of an optimum in the $G({v}_{L})$ curve is due to the aerogel forming cracks when ${v}_{L,PALC}$ exceeds $\sim 10\; mm\,{\bullet\; s}^{-1}$ as the lining then becomes too thick ($i.e.\, >1000\; \mu m$, as reported in [9]). We suspect that the linings of MALCs either also form cracks or become too rough when they become too thick at ${v}_{L,MALC}$ exceeding $\sim 16\; mm\,{\bullet\; s}^{-1}$, but yet cannot substantiate this on the basis of electron microscopic measurements.

Advantageously, the optimal lining thickness depends solely on the wavelength to be guided—and not on the specific analytes dissolved in the aqueous sample. In general, the longer the wavelength of the guided light, the thicker the lining must be (and correspondingly, the higher the lining velocity) in order to prevent lossy interactions—such as absorption, scattering, or refraction—with the capillary wall [18]. Consequently, future optimization of the MALC manufacturing process should aim to reliably produce defect-free linings with minimal refractive index and sufficient thickness to enable efficient light guidance (i.e., optimum Raman signal gain) across a broad wavelength range via total internal reflection.

**Fig. 4:**
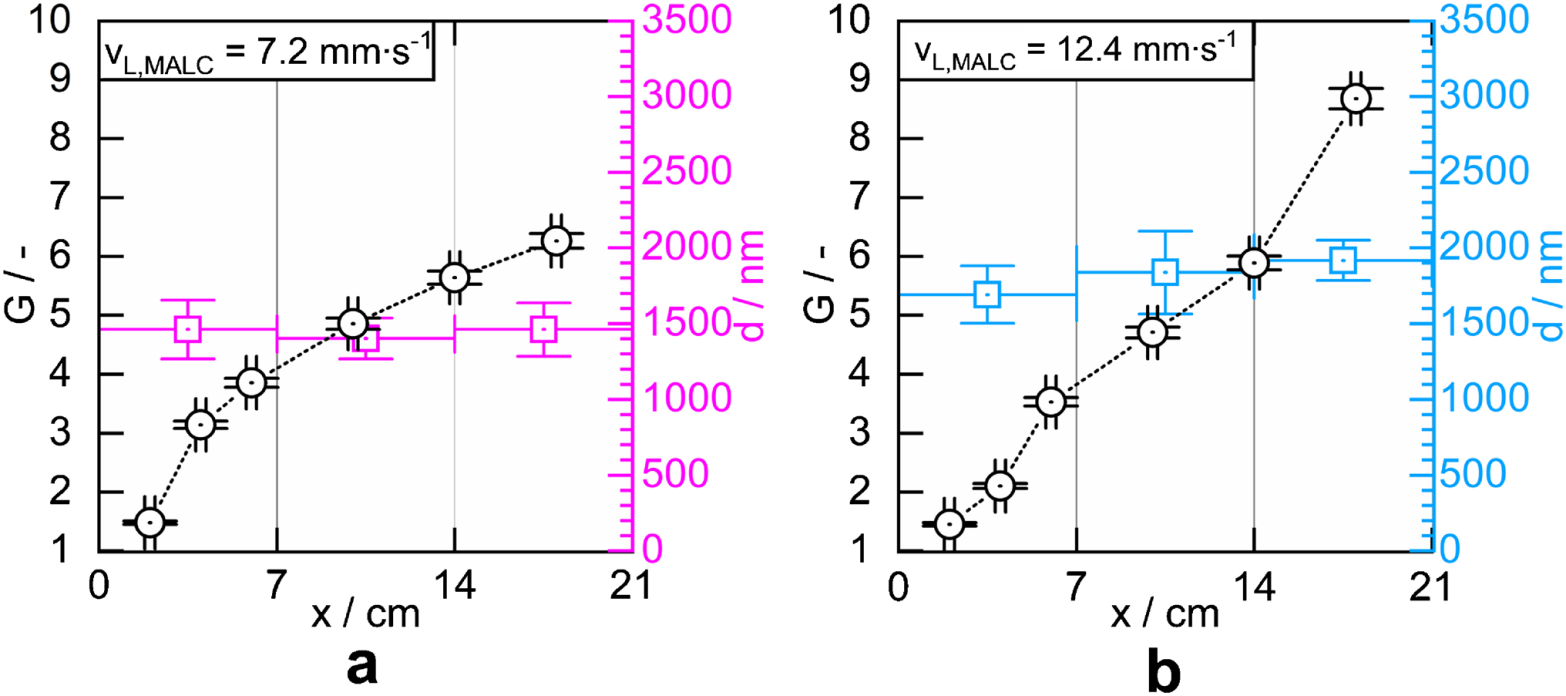
The Raman signal gain G of pure liquid water as a function of the liquid core length x, obtained from two MALCs, fabricated with **a** v_L,MALC_ = 7.2 mm∙s^−1^ and **b** v_L,MALC_ = 12.4 mm∙s^−1^ (the dotted line is a guide for the eye). Also shown are the MALCs’ local aerogel linings’ thicknesses d

The relation between ${v}_{L,MALC}$ and the uniformity of the lining thickness over the length of a MALC was studied using two MALCs produced at lining velocities of $7.2\; mm\,{\bullet\; s}^{-1}$ and $12.4\; mm\,{\bullet\, s}^{-1}$. We measured the Raman signal gain $G$ for liquid water in both MALCs by varying the liquid core length $x$ inside the capillary. It has to be underlined here that when the MALCs or PALCs are immersed completely in the sample, the liquid sample does not automatically penetrate the hollow core. The hydrophobic lining excludes the hydrophilic samples from filling the MALCs or PALCs. The liquid samples have to be sucked with a syringe from a reservoir into the MALCs or PALCs. The length of the liquid core inside the MALCs can be varied simply by pulling more or less sample into the capillary. The 21 cm long MALCs were then broken into three 7 cm segments and then cut into 2–12 mm long pieces. The lining thickness was measured for the different pieces from the different segments using scanning electron microscopy. In Fig. 4, the values $G(x)$ are plotted together with the mean values $d$ of the lining thicknesses measured in the length segments $[0\; cm < x \le 7\; cm],$  $[7\; cm < x \le 14\; cm]$, and $[14\; cm < x \le 21\; cm]$ of the MALCs. The position $x=0\; cm$ defines the top opening of the MALC, in which the excitation laser is coupled into its liquid core. The thickness-error bars represent the single standard deviations from the mean values. The $x$-error bars indicate, respectively, the uncertainty of the length of the liquid core and of the position at which the thickness of the lining was measured.

A comparison of Fig. 4a and b shows that the faster lined MALC achieves a higher maximum $G$, consistent with the $G-{v}_{L,MALC}-$ behavior reported in Fig. 3. Additionally, $G$ of the slower lined MALC in contrast to the faster lined MALC shows a decreasing growth with x. In this regard, the behavior of $G$ over $x$ of the faster lined capillary is more similar to the behavior reported for PALCs (Fig. 11 in [9]).

However, the MALCs shown in Fig. 3, produced at 7.2 and 12.4 mm∙s^−1^, exhibit Raman signal gains of ∼4 and ∼8, respectively, whereas those in Fig. 4a and b, fabricated at the same velocities, show gains of ∼6 and ∼9. This indicates that, at the current stage, reproducibility of MALCs is still challenging. When comparing reproducibility of MALCs and PALCs, however, it is important to note that the viscosity of the PALC-coating dispersion remains stable for several days. This allows uniform lining thickness and consistent Raman signal gain across a batch of PALCs when using the same PALC-coating dispersion and applying the same lining velocity for every single capillary of the batch. In contrast, the viscosity of the MALC-gelling solution changes rapidly within 8 min due to gelation. Therefore, achieving uniform lining thickness and Raman signal gain across a MALC batch requires each gelling solution to have the same viscosity at the moment of coating—a major challenge, because the gelation rate varies slightly between each single GSs, and hence the viscosity of the GSs. In consequence, reproduction of MALCs is significantly more difficult than that of PALCs. However, in situ viscosity measurement could greatly improve MALC reproducibility in the future.

**Fig. 5:**
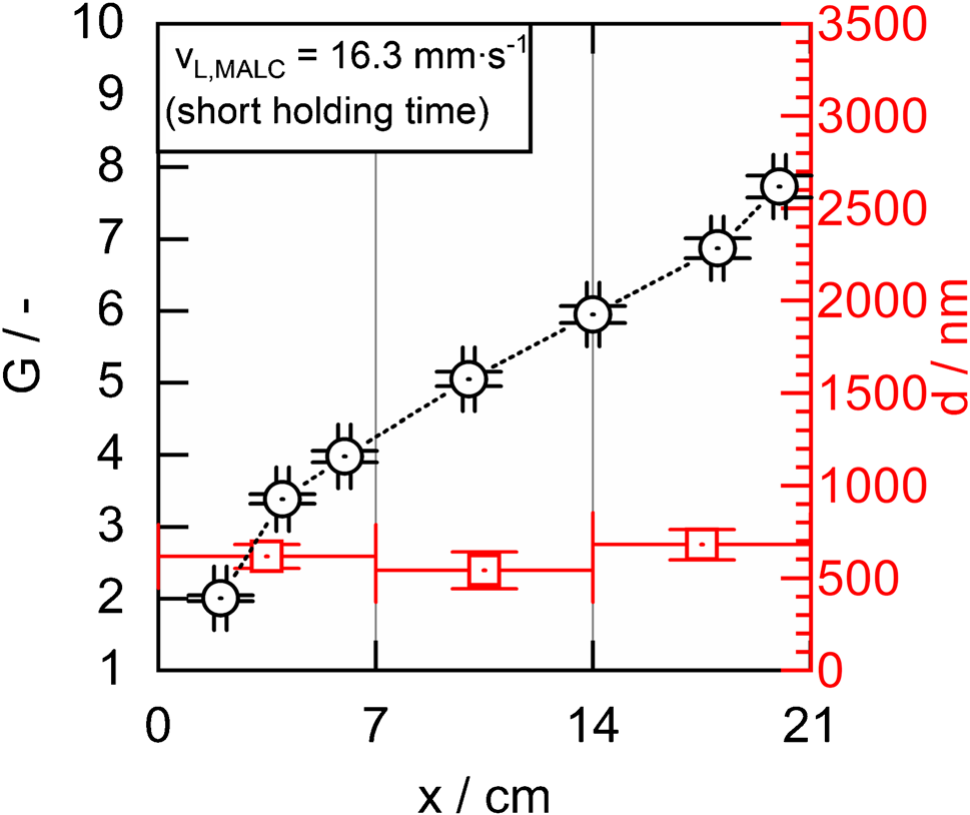
The Raman signal gain G of pure liquid water as a function of the liquid core length x, obtained from a MALC, fabricated with v_L,MALC_ = 16.3 mm∙s^−1^ at a short holding time (the dotted line is a guide for the eye). Also shown is the MALCs’ local aerogel linings’ thickness d

In a further experiment, we reduced the holding time between step 1 and step 2 to just a few seconds (instead of 1 min as described above) while applying a lining velocity of 16.3 mm∙s^−1^ (i.e., higher than 7.2 and 12.4 mm∙s^−1^, applied in Fig. 4a and b). The result (shown in Fig. 5) was a lining featuring a thickness of approximately 600 nm—significantly thinner than the linings in Fig. 4a and b. This is clearly due to the lower viscosity resulting from the reduced holding time and thus the reduced progress of gelation. Surprisingly, however, the Raman signal gain G ≈ 7 of the MALC produced at 16.3 mm∙s^−1^ was not lower but fell between those of the MALCs in Fig. 4a and b (∼6 and ∼9), despite having the thinnest lining. This phenomenon is likely explained by the improved uniformity of the 600 nm thick lining, as indicated by the small error bars in Fig. 5. However, this result motivates us to investigate, in future work, the fabrication of linings composed of multiple thin layers, manufactured at reduced holding time and a lining velocity of 16.3 mm∙s^−1^, with the aim of achieving uniform and sufficiently thick linings that provide optimal Raman signal gain.

Moreover, if a correlation between the local slope of the $G(x)$ curves and the local lining thickness in MALCs can be established in the future, the laborious SEM measurements could be replaced by such a more efficient analysis of the $G(x)$ curves. For instance, reference [19] demonstrates a similar approach to identify local defects in Teflon-AF-based LCWs.

### Application of PALCs and MALCs for Raman signal gain of OxFA process samples

**Fig. 6:**
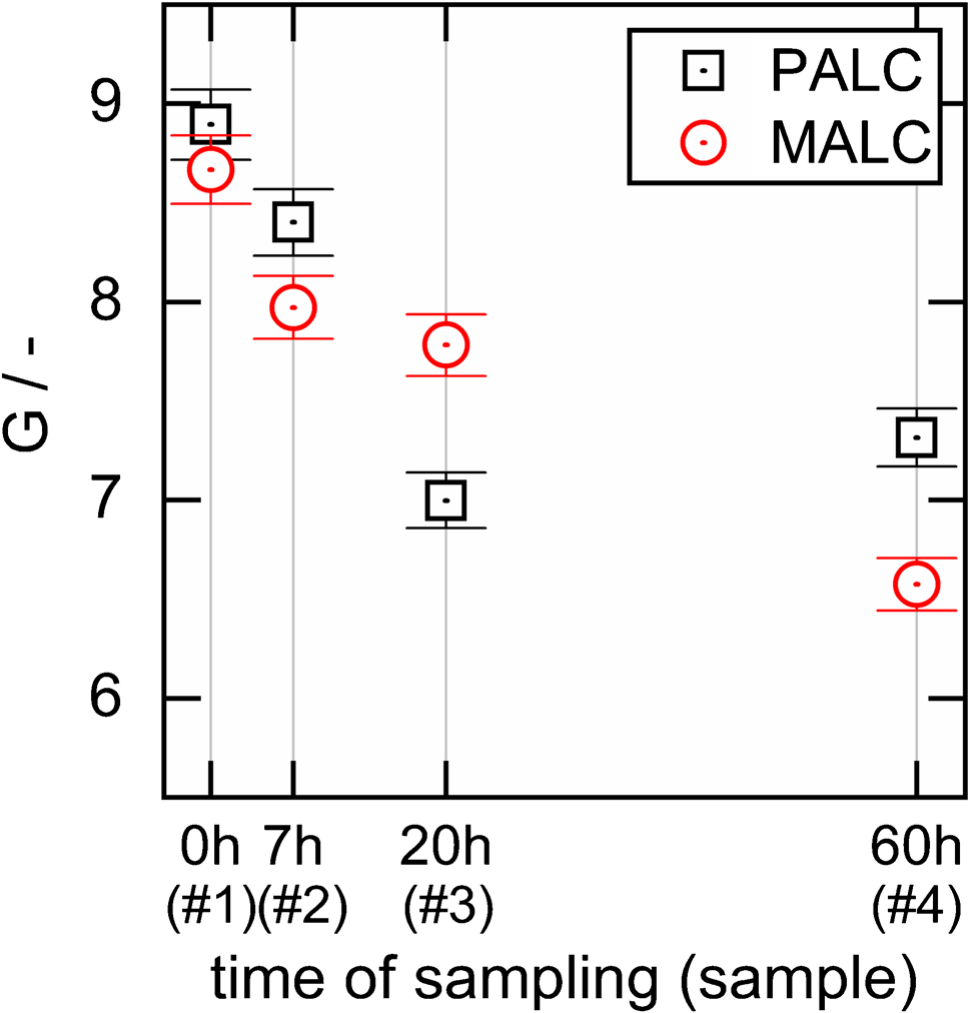
The Raman signal gain G of 12.5 vol.% OxFA samples, obtained from a PALC and from a MALC, plotted against the sample number and the residence time t of the sample on the OxFA process

The Raman spectroscopic analysis of liquid samples with unknown hydrophilicity in PALCs and MALCs should include water dilution to ensure the samples are hydrophilic and do not penetrate the hydrophobic lining. Otherwise, the refractive index of the lining would become quasi identical to the refractive index of the sample and the conditions for TIR would be lost. Therefore, before measuring the Raman signal gain of the OxFA samples in PALCs and MALCs, we diluted samples #1–4 to 12.5 vol%. Figure 6 shows the Raman signal gain of the 12.5 vol% diluted OxFA samples in a PALC and a MALC, which were lined with velocities at ${v}_{L, MALC}$=16.3 mm∙s^−1^ and ${v}_{L, PALC}$=9.8 mm∙s^−1^.

It is obvious that $G$ decreases with increasing sample number # for the PALCs and the MALCs, and that $G$ of all OxFA samples ($G\approx 9-7$) is clearly below the $G$ of pure liquid water ($G\approx 11$, see Fig. 3).

In order to exclude that the decrease in $G$ might be traced back to impurities (side products, catalyst, etc.), which might attenuate the laser or the signal light inside the capillary, we repeated the same experiment in mixtures of pure compounds of glycerol, formic acid, and water in compositions of the OxFA samples. We found the same decrease of $G$ and therefore can exclude the light attenuation effect.

In order to exclude that the decrease in $G$ might be traced back to an increase of the critical angle of TIR, we measured the refractive indices of all samples with an Abbe refractometer at $T=20^\circ C$. The measured indices of refraction are tabulated in Table 3 and are consistently higher than that of liquid water. We therefore can rule out that a decrease in the difference of the refractive indices between the aerogel lining and liquid sample causes the decrease in $G$.

**Table 3:** Measured refractive indices of the 12.5 vol.% OxFA samples #1–4 and of pure liquid water

Sample	Refractive index/-
Water	1.3333 ± 0
12.5 vol% #1	1.3387 ± 0.0001
12.5 vol% #2	1.3372 ± 0.0001
12.5 vol% #3	1.3365 ± 0.0001
12.5 vol% #4	1.3362 ± 0.0001

We also rule out that the decrease in $G$ from samples #1 to #4 is due to abrasion of the aerogel lining during sample filling and emptying, as we measured in the order #4 to #1.

Another possible reason for the decrease in $G$ with sample number is the dissolution of air from the aerogel lining’s pores into the liquid sample. Due to the dissolution the pressure in the lining might decrease, which could eventually suck the liquid sample into the superficial layer of the porous network. In order to test this, we took a new MALC (fabricated at ${v}_{L, MALC}$=21.5 mm∙s^−1^) and measured $G$ of the four 12.5 vol% OxFA samples again. Each sample was then saturated with ambient air for 10 min by bubbling air through the stirred sample using a peristaltic pump, and $G$ was measured again, showing that the decrease in $G$ remained unchanged.

We furthermore agitated the solid aerogel samples from the “Estimation of the density and the refractive indices of monolithic and particle-based silica aerogels” section with the pure and the 12.5 vol% OxFA samples in sealed transparent beakers. The dry solid aerogel flakes still floated on the liquid samples’ surfaces, even after weeks, implying that the liquid samples do not penetrate the porous linings, at least at ambient pressure.

However, when we fill the capillaries with the samples, the samples are sucked at a small vacuum into the MALCs or PALCs. Eventually the hydrophilic samples then might penetrate a short distance into the hydrophobic lining, or few large pores might be filled with the sample. In order to rule out that the OxFA samples, when eventually penetrating into the lining, would irreversibly cause a pore collapse and thus a destruction of the lining, we repeated the measurements shown in Fig. 6 several times with the identical MALC and PALC and obtained the same $G$ values each time, confirming that no destruction of the lining occurs.

**Fig. 7:**
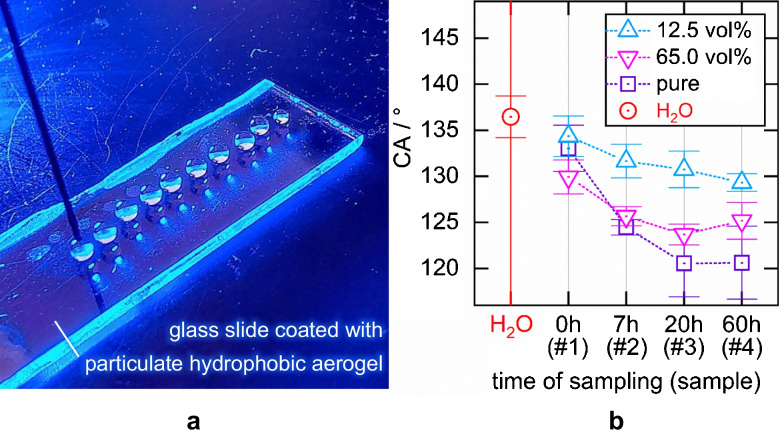
The measurement of the contact angle (CA) between the transparent hydrophobic aerogel surface, pure water, and the OxFA samples (pure, 65 vol% and 12.5 vol%) was carried out in ten repetitions on the same aerogel surface (depicted in **a**). Plotted in **b **is the contact angle (CA) of pure water and of the OxFA samples (see Table 1)

Intuitively, the ability of the hydrophilic samples to penetrate the hydrophobic linings depends on the liquid samples’ ability to wet the aerogels micro- to nanostructured surface. Therefore, we measured the contact angles (CAs) of (i) pure water, (ii) pure OxFA samples, (iii) 65 vol.% OxFA samples, and (iv) 12.5 vol.% OxFA samples on the surface of a hydrophobic silica aerogel-coated glass object slide (according to the “Measuring the contact angle of liquid samples on a silica aerogel surface” section), as depicted in Fig. 7a. The measured contact angles are shown in Fig. 7b. Each contact angle is the mean value of the contact angles of ten individual drops at ten different locations on the object slide (as depicted in Fig. 7a). The error bars show the single standard deviation.

Obviously, pure water features the highest contact angle and thus the least wettability. The contact angles of the three OxFA sample series and therefore the hydrophilicity of the reaction mixture decreases with progress of the OxFA process, caused by glycerol degradation to less hydrophilic carbonyl species. In consequence, the pure samples #3 and #4 feature the lowest hydrophilicity, i.e., the smallest contact angle (≈120° ± 4.5°). The diluted OxFA process samples show a decreasing wettability with dilution. The trend of decreasing CA and $G$ with increasing sample # is likely due to more hydrophobic samples develop a less smooth interface between the liquid core and the aerogel lining by penetrating irregularly into the lining. Of course, a liquid core with a smooth and sharp interface to the air-filled lining better guides light than a liquid core with a non-smooth and degraded interface.

**Fig. 8:**
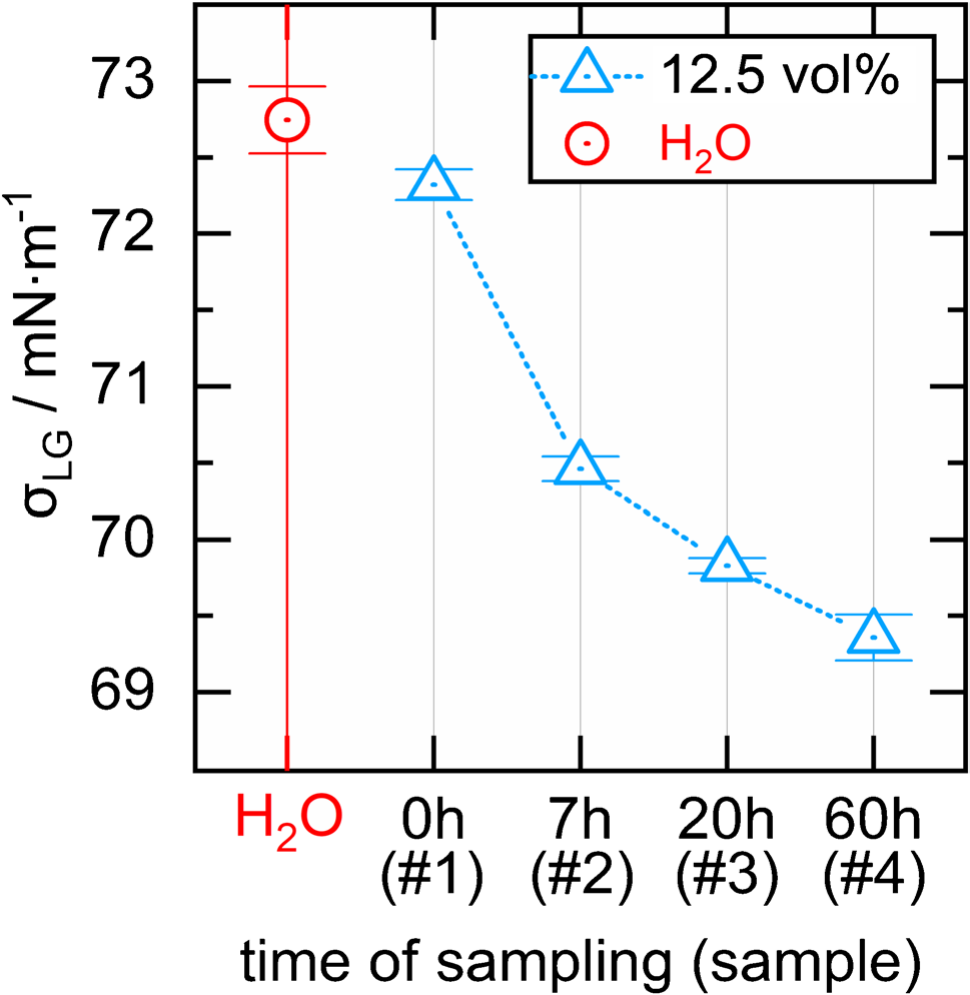
Plot of the interfacial tension ${\sigma }_{LG}$ between water and air and between the 12.5 vol% samples #1–4 and air

Finally, we employed the pendant drop technique as described in the “Measuring the interfacial tension between the liquid samples and air” section for analyzing the interfacial tension between the liquid samples and the air. The interfacial tension represents the systems tendency to provide the smallest possible interfacial area between the sample and the air, even if the interface is non-smooth. We therefore expected systems with a large interfacial tension to develop a smoother interface than systems with a smaller interfacial tension. The results are depicted in Fig. 8 and are the mean values of five single measurements each. The error bars represent the single standard deviation.

The interfacial tension decreases but only slightly with increasing #. However, the decrease in contact angle and the decrease in interfacial tension with increasing # combine, as they have a similar effect on the smoothness of the interface. Together they might cause the worsened light guiding properties of the liquid cores with increasing #. The following consideration is supposed to underline that already a small worsening of the light guiding properties can cause a significant drop in the Raman signal gain: A photon passing through the aqueous core of a $L=20\; cm$ long aerogel-lined capillary with an inner diameter of $D=700\; \mu \text{m}$ and a critical angle ${\alpha }_{c}\approx 50^\circ$ is totally reflected ∼ $L/(D\,\bullet\; tan({\alpha }_{c}))\approx 240$ times at the liquid/air interface. Due to the high number of TIR events, even very small smoothness irregularities of the aerogel lining can have a significant impact on $G$.

### Durability of particulate and monolithic aerogel linings

**Fig. 9:**
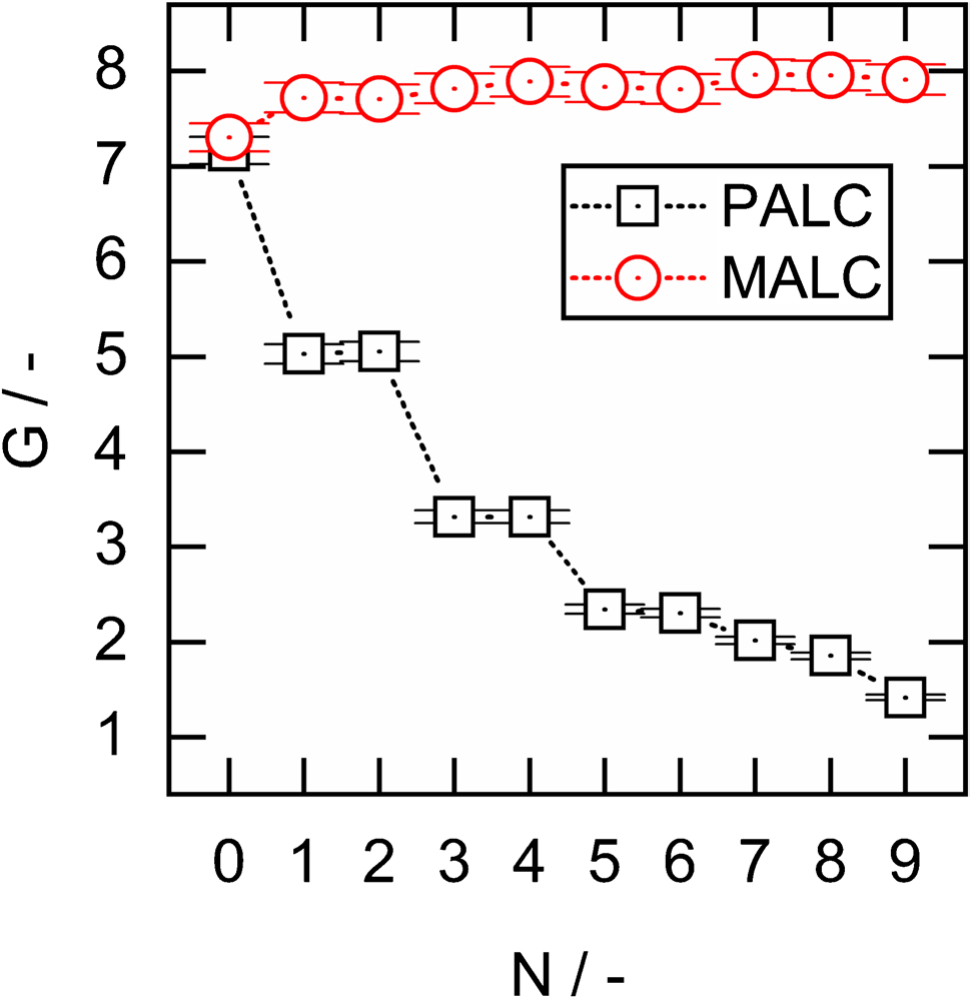
Raman signal gain G of the 12.5 vol% sample #4 achieved by a MALC and a PALC as a function of repetitions N. The PALC and the MALC were flushed by the sample at a flow velocity of ∼ 1 m∙s^−1^

The durability of the aerogel lining determines the lifespan of MALCs and PALCs. A shorter lifespan requires more frequent replacements of the aerogel-lined capillary in a potential Raman monitoring system. Although PALC and MALC aerogel linings are resistant to fouling, scaling and corrosion due to their hydrophobicity, they are still subjected to mechanical stress by the sample flow through the capillaries hollow core. In order to test the flow durability of 21 cm long PALCs and MALCs, we pumped 1 mL of the 12.5 vol% sample #4 through each, applying a flow velocity of $\sim 1\; m\,{\bullet\; s}^{-1}$, recorded the Raman spectra of the liquid sample from the MALC’s and PALC’s and repeated this procedure $N=9$ times. For the first measurement ($N=0$), the liquid sample was filled into the capillaries at a moderate flow velocity of $\sim 0.05\; m\,{\bullet\; s}^{-1}$. The resulting $G(N)$-plot is depicted in Fig. 9 showing that both PALC and MALC provide a comparable Raman signal gain of $G(N=0)\approx 7$.

**Fig. 10:**
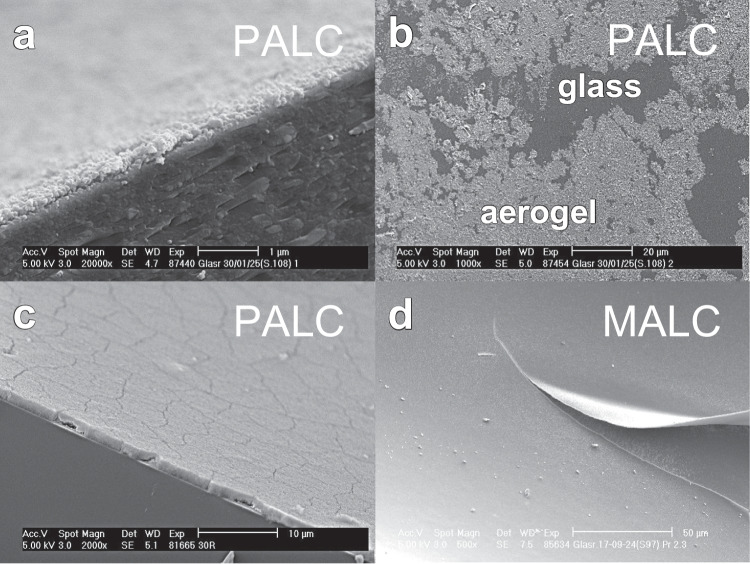
SEM images of **a** the cross-section and **b** the surface of the aerogel lining in a PALC after being flushed *N* = 9 times with the OxFA-60 h sample at 1 m∙s^−1^. The removal of particles in PALCs is facilitated by the tendency for crack formation in the particulate PALC linings (**c**). When MALCs are broken for SEM preparation, the high force can cause the monolithic lining to tear like foil instead of crumbling like the lining of a PALC (**d**)

However, with the increasing number of sample exchanges $N$ conducted at a high flow velocity of $\sim 1\; m\,{\bullet\; s}^{-1}$, the PALC’s Raman signal gain decreases strongly, while the MALC’s one remains constant. The decreasing $G$ of the PALC is likely due to non-covalently bonded aerogel agglomerates being washed away by mechanical stress, as supported by the observation of solid material being flushed out of the PALC and settling on the liquid sample surface inside the cuvette. However, SEM images also confirm that the PALC’s aerogel lining after the experiment features a thickness of only $\sim 0.1\; \mu m$ (see Fig. 10a), whereas the PALC lining thickness was $\sim 1\; \mu m$ at the beginning of the experiment, and is confirmed by the measured Raman signal gain of $G\left(N=0\right)\approx 7$. When examining the surface of the aerogel lining in the PALC, it seems evident that the lining has been extensively washed away due to the high flow velocity of the liquid sample (see Fig. 10b). The washing out of the particulate linings in the PALCs is further facilitated by the tendency for crack formation in the PALCs’ aerogel linings (see Fig. 10c), because at the edges of the cracks the flowing fluid can knock off individual flakes of the particulate lining. Thus, with each further flush, more holes form in the lining, so that after $N=9$ flushes, the PALC provides almost no Raman signal gain any more. In contrast, as the lining in a MALC is a single monolith, no individual parts can detach, and the monolithic linings remain stable even under high fluid mechanical stress. In Fig. 10d, it can be seen that the monolithic lining in a MALC exhibits a completely different mechanical behavior compared to the lining of a PALC; under the high stress that occurs during the breaking of the MALCs for SEM preparation, the MALC’s monolithic lining tears like a foil and does not crumble like the lining of a PALC. However, such a punctual high shear force, which acts on the monolithic lining during sample preparation, does not occur when a liquid sample flows through the hollow core of the MALC. Thus, the MALC showed unchanged performance even after $N>30$ sample exchanges, presumably because the lining sticks tightly on the inner surface of the capillary wall – just like a slightly elastic foil. This high robustness makes the MALC a significantly more durable and safe product compared to the PALC and probably justifies the MALC’s higher production effort.

## Conclusions

We have presented the manufacturing of monolithic aerogel-lined capillaries (MALCs), serving as liquid core waveguides which gain the Raman signal of liquid aqueous samples (e.g., from the OxFA process) and keep up with the previously presented particulate aerogel-lined capillaries (PALCs). However, the Raman signal gain of both MALCs and PALCs seems to be a function of the wettability of the lining and the surface tension of the sample. This functionality must be subject to further investigations. As MALCs and PALCs are chemically resistant and compatible with acidic aqueous-organic solutions, future studies will investigate the relationship between wettability and Raman signal gain in similar samples, such as those from biotechnological processes [20–22]. Special attention will be given to the impact of living organisms or enzymes dissolved in the reaction medium on wettability. Additionally, the potential of multiple hydrophobization treatments to increase the content of hydrophobic methyl groups in the lining—thereby enhancing hydrophobicity and reducing wettability—will be examined.

However, MALCs offer a distinct advantage over PALCs due to their monolithic, crack-free linings, which provide high mechanical stability even when the liquid sample in the hollow core is exchanged rapidly and frequently. This enhanced robustness increases the reliability of MALCs under demanding conditions and makes them particularly well suited for industrial process monitoring. While their fabrication may be more complex than that of PALCs, this drawback may be offset by their improved performance and durability in critical applications.

Since the optimal lining thickness in MALCs (and PALCs) depends solely on the TIR-guided wavelength—not on the specific analytes—future efforts will focus on reliably manufacturing defect-free linings with minimal RI and sufficient thickness to ensure efficient light guidance and thus optimal Raman signal gain across a broad wavelength range. However, at the current state, achieving reproducible lining thickness in MALCs is challenging due to the rapid increase in the gelling sol’s viscosity. But, implementing in situ viscosity monitoring during gelation could address this challenge in future and significantly improve the reproducibility of MALCs. Furthermore, if the lining thickness can be derived from the slopes of the G(x) curves in future, the number of necessary SEM analyses will be greatly reduced, making the manufacturing of MALCs (and PALCs) significantly more efficient. Future tasks will also be the improvement of the uniformity of the monolithic linings thickness (probably by optimizing gelation-pH and therefore gelation time [17]) and the manufacturing of longer MALCs.

## Data Availability

Data underlying the results presented in this paper are not publicly available at this time but may be obtained from the authors upon reasonable request.

## Declarations

### Patent

The technique presented in this publication for the manufacturing of a monolithic silica aerogel-lined capillary is covered by the German patent application No. 10 2025 113 384.0.
